# Influenza Vaccination is Associated with Lower Risk of Acute Coronary Syndrome in Elderly Patients with Chronic Kidney Disease

**DOI:** 10.1097/MD.0000000000002588

**Published:** 2016-02-08

**Authors:** Chang-I Chen, Pai-Feng Kao, Mei-Yi Wu, Yu-Ann Fang, James S. Miser, Ju-Chi Liu, Li-Chin Sung

**Affiliations:** From the Taipei Cancer Center (C-IC, Y-AF), Cancer Center, Wan Fang Hospital (C-IC, Y-AF), Department of Healthcare Administration (C-IC), Division of Cardiology, Department of Internal Medicine, Shuang Ho Hospital (P-FK, J-CL, L-CS), Division of Nephrology, Department of Internal Medicine, Shuang Ho Hospital (MYW), College of Medical Science and Technology (JSM), and Department of Internal Medicine, School of Medicine, College of Medicine (P-FK, JCL), Taipei Medical University, Taipei, Taiwan; City of Hope National Medical Center, Duarte, CA (JSM).

## Abstract

Elderly patients with chronic kidney disease (CKD) are at a higher risk of hospitalization for cardiovascular diseases (CVD). Previous studies have showed that influenza vaccination could reduce the risk of recurrent major cardiovascular events in patients with CVD. However, the effects of influenza vaccination on the reduction of first hospitalizations for acute coronary syndrome (ACS) in elderly patients with CKD remain unknown.

We conducted a cohort study using data from the Taiwan Longitudinal Health Insurance Database 1997 to 2008. This cohort study comprised elderly patients (ages ≥55 years) with a recorded diagnosis of CKD (n = 4406) between January 1, 1999, and December 31, 2007. Each patient was followed up until the end of 2008. To minimize the selection bias of vaccine therapy, a propensity score adjustment was applied. The hazard ratio (HR) and 95% confidence interval (CI) for the association between the influenza vaccination and the occurrence of first hospitalization for ACS was evaluated by Cox proportional hazards regression. We further categorized the patients into 4 groups according to their vaccination status (unvaccinated, and total number of vaccinations: 1, 2–3, and ≥4).

We found that elderly CKD patients without prior CVD history receiving influenza vaccination exhibited a lower risk of hospitalization for ACS (adjusted HR = 0.35, 95% CI 0.30–0.42; *P* < 0.001). We observed consistent protective effects regardless of age groups (55–64, 65–74, and ≥75), gender, and seasonality of influenza. When the patients were stratified according to the total number of vaccinations, the adjusted HRs for first ACS hospitalization were 0.62 (95% CI 0.52–0.81), 0.35 (95% CI 0.28–0.45), and 0.13 (95% CI 0.09–0.19) for patients who received 1, 2 to 3, and ≥4 vaccinations. There was a significant trend of decreasing risk of ACS hospitalization with an increasing number of vaccinations.

The results of our observational study could strengthen the annual vaccination policy and physicians should be aware of missed opportunities to vaccinate elderly patients with CKD against influenza. The potential public health impact of influenza vaccination, particularly in the elderly CKD patients without a history of CVD, who are at risk for ACS, should be further explored.

## INTRODUCTION

Chronic kidney disease (CKD), a chronic inflammatory disease, is more prevalent in Taiwan than in most other countries since 2000.^1^ A large cohort study based on 462,293 adults in Taiwan showed that the prevalence of CKD is high (11.9%) in adults and the prevalence is even higher (37.2%) among the elderly.^2^ Previous studies show that CKD is associated with high morbidity and mortality, which are mainly associated with cardiovascular diseases (CVD).^3^ Patients with CKD have higher rate of cardiovascular mortality, mostly associated with coronary artery disease (CAD) than age-matched controls without CKD.^4^ The initial clinical presentation of CAD is often acute coronary syndrome (ACS), which tends to be more complicated and has a higher mortality risk in CKD population.^5^ The possible explanations of this poor outcome are the high rates of advanced age, diabetes, hypertension, chronic inflammation, and coronary artery calcification.^5^

During influenza epidemics, acute respiratory tract infections may trigger ACS.^6–8^ Previous studies have shown that vaccination for influenza can reduce the risk of recurrent major cardiovascular events in patients with stable CAD or ACS.^9,10^ Among elderly individuals, those with CKD are at a higher risk for developing serious influenza-related complications given their altered immune response and persistence.^11,12^ The serious complications of influenza can cause hospitalization or even death in this group of patients. Influenza vaccination appears to be associated with lower mortality and hospitalization rates among patients with CKD or end-stage renal disease (ESRD),^11,13–15^ which is likely to benefit from prevention of respiratory-related or cardiovascular complications. Both CKD and ACS are growing public health issues because population aging contributes to the increasing incidence of both diseases. However, data regarding the protective effect of vaccine for developing ACS in elderly patients with CKD is limited. To clarify the potential protective benefit of influenza vaccination on hospitalization for first ACS in elderly Taiwanese patients with CKD, we conducted a population-based cohort study by using reimbursement claims data from Taiwan's National Health Insurance Research Database (NHIRD).

## METHODS

Taiwan initiated its National Health Insurance (NHI) program in 1995 to provide comprehensive health insurance coverage for all of Taiwan residents. Currently, almost 99% of the more than 23 million enrollees are covered under the NHI. This study used data from the Taiwan NHIRD 1997 to 2008 consisting of 1,000,000 randomly sampled people. There were no statistically significant differences in sex, age, or healthcare costs between the sample group and all enrollees. The Taiwan Center of Disease Control typically defines the influenza season as the period between October and next March.^16^ Influenza viral activity usually begins to rise in late October, and peaks during the end of December and March in the following year. The information obtained from the database was entirely anonymous, and the study was approved by the NHIRD research committee and the Joint Institutional Review Board of Taipei Medical University (TMU-JIRB No. 201311026).

**FIGURE 1:**
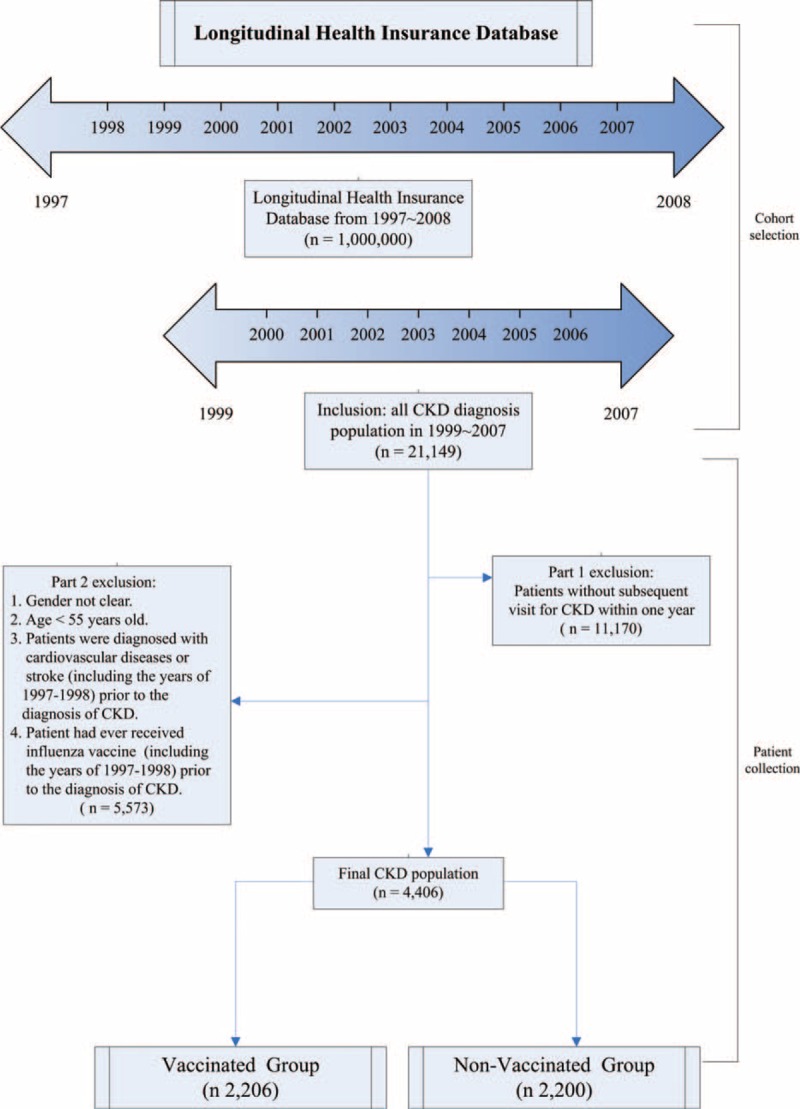
Flowchart demonstrates the selection criteria and process of CKD population. CKD = chronic kidney disease.

The study cohort comprised all patients diagnosed with CKD, based on International Classification of Disease, Ninth Revision, Clinical Modification (ICD-9-CM) code 585, who visited healthcare facilities in Taiwan over a 9-year period (n = 21,149) from January 1, 1999 to December 31, 2007. All patients without a subsequent outpatient visit, emergency department visit, or hospitalization for CKD within 12 months of first presentation were excluded (n = 11,170; Figure 1). We excluded individuals who are younger than 55 years old (n = 3911) or patients with unclear gender (n = 2; Figure 1). Additionally, we used a 2-year washout period (1997–1998) to ensure that all CKD cohorts had no prior cardiovascular comorbidities. Therefore, patients who had a diagnosis of ACS or angina pectoris (n = 1123), stroke (n = 772), or heart failure (HF; n = 647) between January 1, 1997 and the date of CKD diagnosis were excluded. In Taiwan, influenza vaccination has been free of charge and recommended for the high-risk elderly population since 1998, and for all adults older than 65 years since 2001.^17^ The vaccination status was identified by ICD-9-CM code V04.8 and the use of vaccine (confirmed by the drug codes). All individuals with influenza vaccination between January 1, 1997 and the date of CKD diagnosis were excluded (Figure 1). The index date of the vaccinated group was defined as the date of the first influenza vaccination. Furthermore, patients were excluded from the analysis if they had a history of ACS hospitalization before the index date. Patients who never received influenza vaccine between 1997 and 2008 were designated as the unvaccinated group. The index date of the unvaccinated group was defined as the date of CKD diagnosis during the entry period. Each patient was tracked for the Charlson Comorbidity Index (CCI), which is suitable for general medical inpatient populations and is a useful prognostic predictor in CKD patients.^18,19^ A propensity score (PS) was derived using a logistic regression adjustment to balance the covariates that predict receiving the vaccine therapy in the 2 groups. This method is used in observational studies to identify true causal relationship and reduce selection bias.^20^ The covariates used to calculate the PS were gender, age, comorbidities (CCI, pneumonia, hypertension, diabetes, dyslipidemia, anemia), cardiac arrhythmia, regular dialysis, monthly income (0, NT$1–15,840, NT$15,841–25,000, NT$ ≥25,001; NT$ represents New Taiwan Dollars), urbanization level (urban, suburban, and rural), and geographic location of residency (Northern, Central, Eastern, and Southern Taiwan).^21,22^ Each patient was followed up from the index date of each group until the development of hospitalization for acute myocardial infarction (MI) (ICD-9-CM codes 410.xx and 411.xx) or angina pectoris (ICD-9-CM codes 413.xx and 414.xx) with percutaneous coronary intervention (PCI) or coronary artery bypass grafting (CABG) procedures, death, withdrawal from the insurance, or December 31, 2008, whichever occurred first. The patients were followed for a minimum of 12 months to a maximum of 10 years. Because the vaccine does not take effect within the initial 2 weeks of administration, we excluded patients for whom the duration between vaccination and first ACS hospitalization was shorter than 2 weeks.^23^ Data were collected from the claims records of discharged patients and analyzed.

### Statistical Analysis

Chi-squared analyses were used to compare the differences between the vaccinated and unvaccinated groups in the relationships among comorbidities, demographic variables, and socioeconomic status. The hazard ratio (HR) and 95% confidence interval (CI) for the association between the influenza vaccination and the first hospitalization for ACS were examined using Cox proportional hazards regression analysis. In addition, the seasonal effect of vaccination on hospitalization for ACS was evaluated. The cumulative rate of ACS development in the 2 groups was estimated by using the Kaplan–Meier analysis. To examine the effect of the total number of vaccines on the cumulative incidence of ACS hospitalization, we categorized the patients into 4 groups according to vaccination status (unvaccinated, total number of vaccinations: 1, 2–3, and ≥4). These data were stratified according to the patients’ age and sex and the requirements for dialysis. Statistical analyses were performed using SPSS 19.0 and SAS 9.2 software. Two-tailed *P* < 0.05 was considered statistically significant.

## RESULTS

**FIGURE 2:**
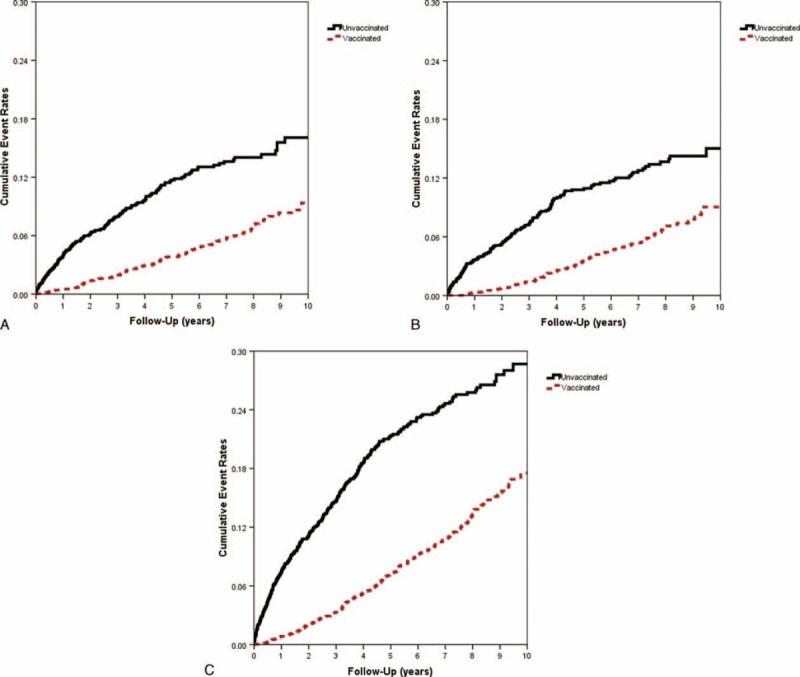
Cumulative hospitalization rates for ACS in elderly patients with CKD in Taiwan (n = 4406), stratified according to the influenza vaccination status during (A) the influenza season (log-rank test, *P* < 0.001), (B) the noninfluenza season (log-rank test, *P* < 0.001), and (C) all seasons (log-rank test, *P* < 0.001). ACS = acute coronary syndrome, CKD = chronic kidney disease.

**FIGURE 3:**
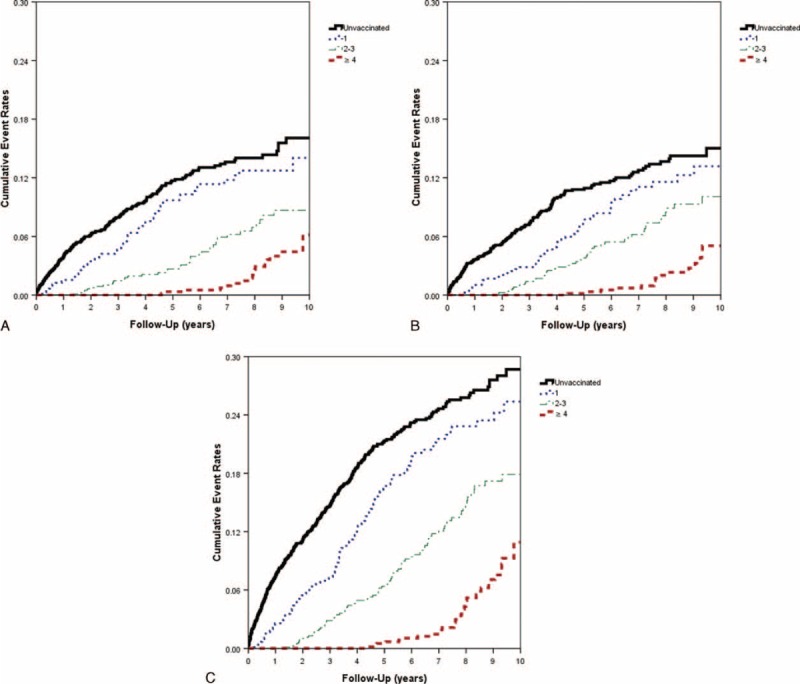
Cumulative hospitalization rates for ACS in elderly patients with CKD in Taiwan (n = 4406), stratified according to the total number of vaccinations during (A) the influenza season (log-rank test, *P* < 0.001), (B) the noninfluenza season (log-rank test, *P* < 0.001), and (C) all seasons (log-rank test, *P* < 0.001). ACS = acute coronary syndrome, CKD = chronic kidney disease.

The eligible study population consisted of 4406 individuals in the CKD cohort. There were 2206 patients in the vaccinated group and 2200 in the unvaccinated group (Table 1). The unvaccinated group exhibited a higher prevalence of certain preexisting medical comorbidities, including high CCI, hypertension, diabetes, dyslipidemia, dialysis, and anemia than the vaccinated group did before the PS adjustment. In addition, significant differences between the 2 groups were observed in the distributions of age, gender, socioeconomic status, and urbanization level (Table 1). Table 2 shows that the rate of hospitalization for ACS after adjusting potential confounders was significantly lower in the vaccination group (adjusted HR = 0.35, 95% CI 0.30–0.42; *P* < 0.001) than in the unvaccinated group. We observed similar protective effects in both genders and all elderly-age groups (55–64, 65–74, and ≥75 years). Influenza vaccination significantly reduced the risk of ACS hospitalizations in elderly patients with CKD irrespective of influenza seasonality (Table 2). The Kaplan–Meier estimates of cumulative ACS event rates in the unvaccinated control were significantly higher as compared to the vaccinated group (log-rank test, *P* < 0.001; Figure 2A–C). When the patients were stratified according to the total number of vaccinations, the adjusted HRs for first ACS hospitalization were 0.62 (95% CI 0.52–0.81), 0.35 (95% CI 0.28–0.45), and 0.13 (95% CI 0.09–0.19) for patients who received 1, 2 to 3, and ≥ 4 vaccinations during the follow-up period (all *P* < 0.001), respectively (Table 3). The cumulative hospitalization rates for ACS, stratified by the total number of vaccinations are shown in Figure 3A–C.

**TABLE 1:**
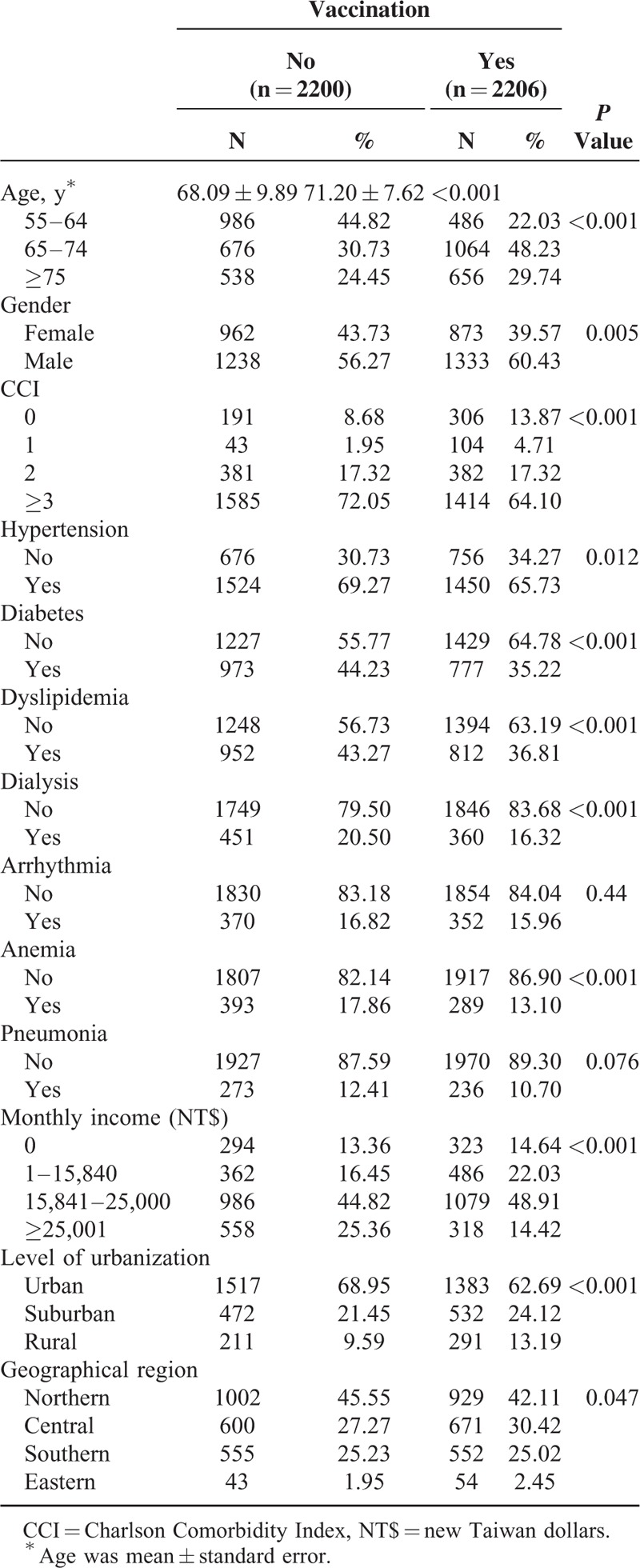
Comparisons in Demographic Characteristics Between Cohorts With and Without Vaccination

**TABLE 2:**
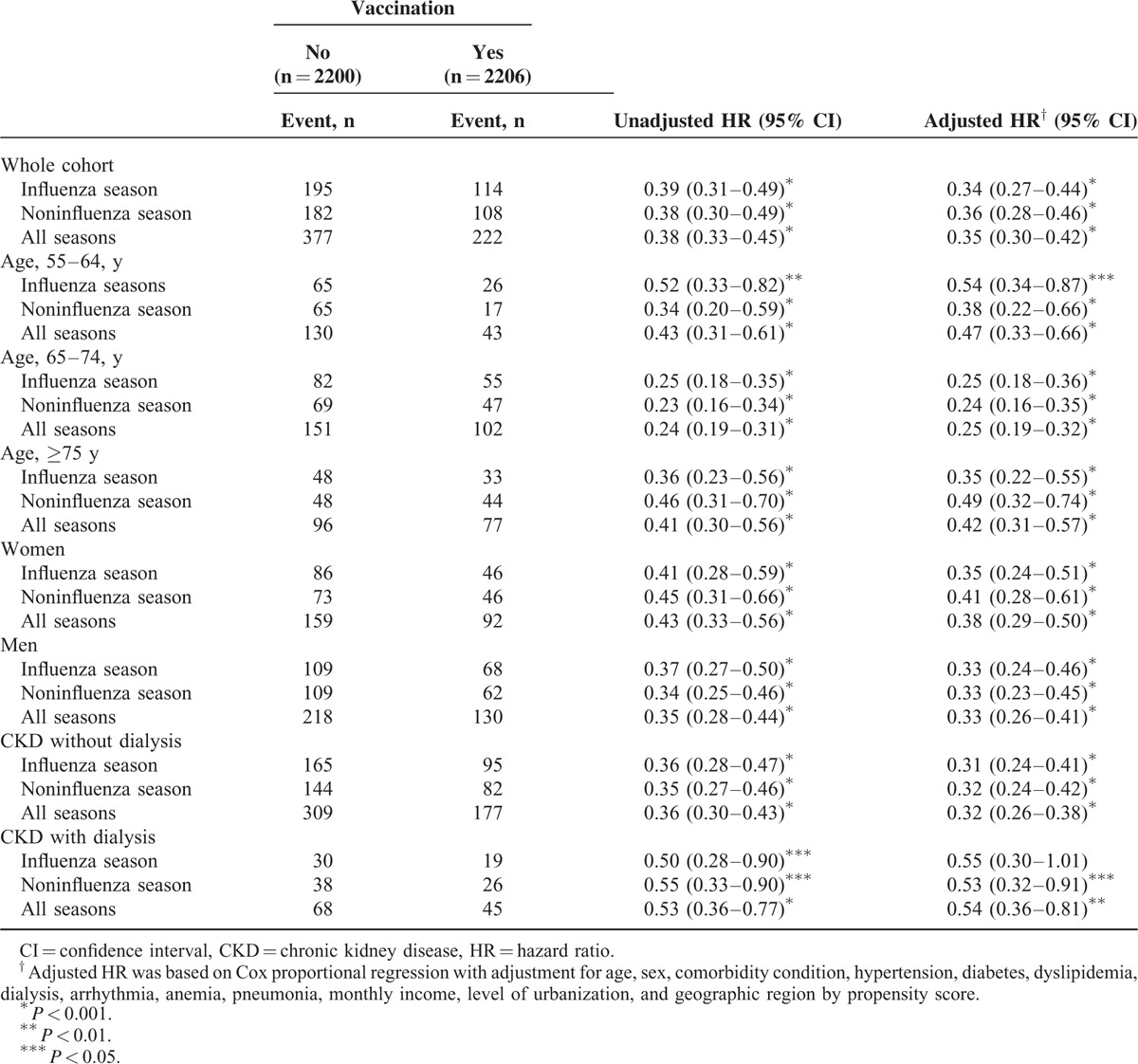
Risk of Hospitalization for Acute Coronary Syndrome in Patients With Chronic Kidney Disease Stratified by Vaccination Status During Influenza and Noninfluenza Seasons

**TABLE 3:**
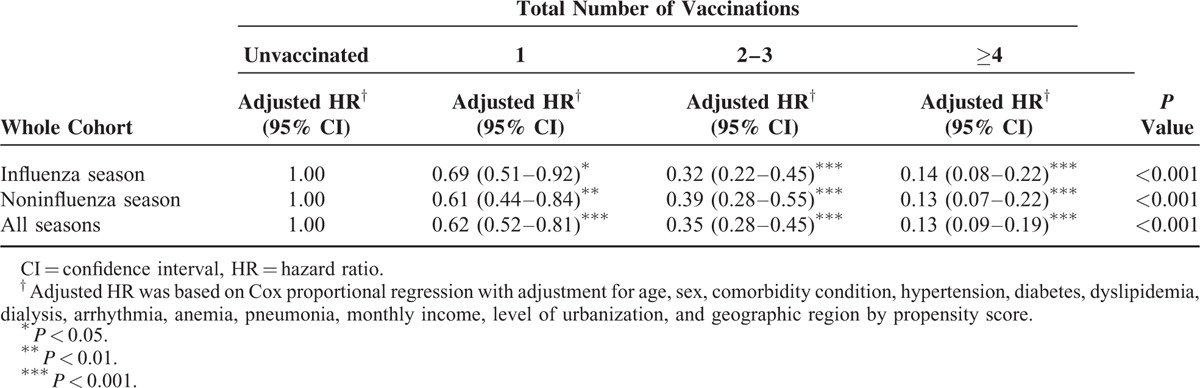
Risk of Hospitalization for Acute Coronary Syndrome in Patients With Chronic Kidney Disease Stratified by the Total Number of Vaccinations During Influenza and Noninfluenza Seasons

## DISCUSSION

In this study, we found that elderly CKD patients without prior CVD history, who had received influenza vaccination, were associated with a lower hospitalization rate due to newly diagnosed ACS.

Several previous reports suggest that CKD increases the risk of cardiovascular morbidity and mortality.^3,4,24,25^ CKD patients without prior evidence of CVD were 60% more likely to develop CVD during the subsequent year than non-CKD patients.^26^ Further, the risk for CAD increases gradually with the decline of renal function and approximately 50% of the mortality in individuals with ESRD is due to cardiovascular events.^4,5^ Besides, United States Renal Data System analyses show hospitalization rates for ACS, arrhythmias, and HF were 2 to 7 times higher for Medicare patients with CKD than for those without CKD.^24^ There are several possible explanations for the relation between CKD and increased risks of CAD. Except for shared common etiologic factors between CKD and CAD (such as diabetes, hypertension, and dyslipidemia), the reduced renal function is associated with chronic inflammation, elevated plasma homocysteine, increased proinflammatory cytokines due to both increased production and decreased renal clearance, enhanced thrombogenicity, abnormal apolipoprotein levels, anemia, neurohormonal activation, increased arterial calcification, endothelial dysfunction, and excessive oxidative stress.^4,5,25,27^ The National Kidney Foundation and American Heart Association have classified CKD as a CVD risk equivalent and previous study revealed the cardiovascular mortality in elderly CKD patients (≥65 years) is as high as in patients with preexisting MI (HR = 0.8, 95% CI 0.6–1.1).^5,28^

Influenza is a major cause of hospitalization and mortality for elderly patients, with more than 20,000 annual influenza deaths worldwide.^29^ The morbidity and mortality of influenza is higher among the elderly, immune-compromised subjects and those with chronic diseases such as CKD or CVD.^11,30^ Systemic inflammation caused by influenza infection may lead to vascular events due to immune activation of atherosclerotic plaque, endothelial damage, impaired vasodilatation, and enhanced thrombogenicity.^30,31^ Although vaccinations are 50% to 60% effective at preventing influenza in the elderly, vaccinations may be up to 80% effective at preventing the serious cardiovascular complications of influenza.^32^ In spite of this, only 45% of elderly ages 65 years or older receive influenza vaccination annually in 2008 in Taiwan.^33^

Patients with CKD are more likely to be hospitalized for infection-related complications than patients without CKD.^11,34–36^ Among Medicare beneficiaries ages ≥66 years old, patients with CKD have higher rates of hospitalization for pneumonia and sepsis compared with patients without CKD.^34^ Potential risk factors for infection among patients with CKD include advanced age, more coexisting disorders, hypoalbuminemia, malnutrition, and anemia.^34^ The 2 major complications (ACS and infection) in CKD patients appear to be closely linked. Infection and underlying CKD are both associated with inflammatory status that may contribute to the physiopathology of atherosclerosis and the development of ACS.^6,7,9,10,36,37^ The possible mechanisms of triggering of ACS by acute infections in CKD patients are: increased and persistent vascular inflammation, increased pro-coagulant conditions, vasoconstriction, triggering of an autoimmune process, increased biomechanical stress, increased platelet activation, endothelial dysfunction, increased metabolic demand, hypoxemia, and hypotension.^5,37^

To minimize the risk of infection and CVD complications, care of CKD patients should include prophylactic preventions such as the administration of a seasonal influenza vaccine. Phrommintikul et al reported a prospective randomized trial including 439 patients with prior ACS that major cardiovascular events (death, ACS, HF, and stroke) occurred less frequently in the influenza vaccine group (n = 221) than in the control group (n = 218) (9.5% vs 19.3%, HR = 0.67 [95% CI 0.51–0.86]; *P* = 0.005),^10^ which shows the efficacy of influenza vaccine against ACS. The vaccine may exert this protective effect either by promoting immunity to the virus or by reducing inflammation.^10,11,17,24^ It seems reasonable that influenza vaccination is also effective in CKD patients, who have equivalent CVD risk as in patients having prior ACS.^28^ Because CKD is listed as an indication for an influenza vaccine in the WHO/Europe 2012/2013 recommendations^38^ and are prone to complications of influenza infection, evaluating the efficacy of vaccination in this patient group was necessary. Several studies had showed that influenza vaccination can reduce the risk of recurrent major cardiovascular events in patients with CKD, but all of the study group patients had ESRD and concomitant CVD with the incidence about 6% to 48.3%.^9,11,13–15,17,35^ Thus, evidence on the effectiveness of influenza vaccination in the primary prevention of CVD in CKD patients is lacking.^39^ We found that vaccination can provide protection for CVD in elderly patients with CKD. Our study strengthened previous reports that influenza vaccine is beneficial in preventing cardiovascular events in ESRD patients. Furthermore, our data also showed the same beneficial effect in CKD patients without dialysis, which is rarely present in the literature. In the present study, the protective effects of vaccines were nearly identical during the influenza and noninfluenza seasons. Our observations are compatible with 1 earlier report regarding the similar protective effect in patients with CVD during the influenza and noninfluenza seasons.^40^ The exact reason for this phenomenon is unknown. One possible explanation is that vaccines protection during the noninfluenza season may be due to a carryover effect.^40^ An alternative explanation is that the severity, timing, and length of influenza season vary each year in Taiwan. Earlier studies showed an impaired response to influenza vaccination in CKD/ESRD patients, but more recent report showed that the vaccine is still able to induce immunity in a significant number of patients.^35,41^ However, more rapid loss of proliferating cells and specific T-cell memory over time in CKD/ESRD patients may represent a sign of relative immunodeficiency.^41^ Regarding the persistence of immunogenicity after vaccination, the proportion of persons who retained protective levels of anti-influenza antibody declined within 1 year.^42^ We recommend annual vaccination, because our results indicated that the protective effect intensifies as the total number of annual vaccinations increases.

There are several limitations. First, the diagnoses of ACS and CKD were identified based on the ICD-9-CM codes and the diagnostic accuracy may be a concern. Because ACS is a clinical diagnosis, we defined ACS hospitalization according to discharge claims data and procedural codes to ensure that the diagnoses were reliable and valid. Moreover, the lack of laboratory values in NHIRD makes it impossible to clarify the CKD stage, measure influenza infection, and circulating viral strains. Second, because of the inherent limitations of the NHIRD, information about CAD risks such as records of smoking habits, family history, body mass index, lifestyle, health behavior, or unhealthy habits were unavailable. However, we think the distribution of the unmeasured confounding factors between 2 groups was similar. In addition, because the study cohort included only Taiwanese elderly patients, meaning that the results may not be generalizable to other populations and persons <55 years of age. Finally, although we attempted to include all the possible confounding factors, the possibility of residual bias persists. The clinical relevance of this study needs to be further validated by larger scale prospective randomized trials.

## CONCLUSION

The results of our study provide clinically important evidence suggesting that annual influenza vaccination is associated with a lower risk of hospitalization for ACS in elderly patients with CKD. Physicians should be aware of missed opportunities to vaccinate elderly patients with CKD against influenza.
